# BioTM Buzz Volume 4, Issue 2

**DOI:** 10.1002/btm2.10135

**Published:** 2019-06-12

**Authors:** Aaron C. Anselmo

**Affiliations:** ^1^ Division of Pharmacoengineering and Molecular Pharmaceutics Eshelman School of Pharmacy, University of North Carolina at Chapel Hill Chapel Hill NC

## INTRODUCTION TO SPECIAL ISSUE

This special issue of *Bioengineering & Translational Medicine* (Volume 4, Issue 2) features articles that address the main topics at the 2018 ECI Nanotechnology in Medicine conference, held from June 5–9 in Albufeira, Portugal. The conference chairs, Dr. Millicent Sullivan of the University of Delaware and Dr. Josué Sznitman of Technion‐Israel Institute of Technology, have served as Guest Editors for this special issue. This is the first of two special issues dedicated to the ECI Nanotechnology in Medicine conference.

Nanotechnology is increasingly prominent in medicine for a broad range of applications ranging from evaluation of cellular and molecular interactions determining drug efficacy to the downstream development of technologies for disease characterization, detection, and treatment. Despite its enormous promise, few nanotechnology platforms have translated from bench to bedside and proven their clinical utility. To address this roadblock, the ECI Nanotechnology in Medicine conference series was designed as a diverse discussion forum aimed at (a) providing a deepened mechanistic understanding of the interactions and responses of nanotechnologies within biological systems at multiple scales; and (b) showcasing and evaluating cutting‐edge nanotechnologies for the early detection, imaging, and treatment of human diseases. The key thematic focus area at the 2018 meeting, revolving around the topic of “bridging translational in vitro and in vivo interfaces,” was the challenges in design and integration of effective platforms for nanotechnology evaluation, ranging from cellular and subcellular analyses, to cell culture environments and 3D models of the cell niche, to lab‐on‐a‐chip systems for analyzing interactions between multiple tissue or organ systems. The conference brought together a broad range of academic and industry scientists covering the interdisciplinary fields of biology, chemical and bioengineering, material science, physics, and others.

## TWO DRUGS ARE BETTER THAN ONE

Combination chemotherapy approaches are widely used to treat many solid tumors in the clinic and have been shown to improve cancer patient survival. These approaches, however, typically leverage combinations of at least two chemotherapies at their maximum tolerated doses and not at ratios that are tuned specifically for that patient or optimized for their tumor/cancer. As such, opportunities for optimization of doses, specifically in identifying synergistic pairs of chemotherapies at molar ratios below their individual maximum tolerated doses, exist. In this issue of Bioengineering & Translational Medicine, a team of engineers led by Professor Samir Mitragotri in the John A. Paulson School of Engineering and Applied Sciences and the Wyss Institute of Biologically Inspired Engineering at Harvard University, describe a systematic approach to evaluate two topoisomerase I inhibitors (doxorubicin and camptothecin) toward identifying synergistic doses of the drug against triple negative breast cancer. The leading formulation, which was fivefold lower than the maximum tolerated doses for the individual drugs doxorubicin and camptothecin, exhibited over 10‐fold decrease in tumor volume as compared to saline controls in an in vivo MDA‐MB‐231 triple negative breast cancer model. Furthermore, the authors show that combination chemotherapies initiated an anti‐tumor immunogenic response in a 4T1 in vivo model wherein tumor associated macrophages were polarized toward their anti‐cancer phenotype. Broadly, this paper provided a systemic and rational approach toward utilizing a known anti‐cancer drug (camptothecin) that has been limited in clinical use due to high toxicity, in a low‐dose combination formulation that exhibits significantly higher efficacy and lower toxicity.

DOI: 10.1002/btm2.10129

## VACCINE STORAGE AND DELIVERY FROM MICRONEEDLES

Many vaccines, especially live‐attenuated vaccines, require cold‐chain storage which can limit vaccination in the developing world. The administration of the vaccines in the developing world can also represent a challenge, especially if the vaccines need to be modified or re‐formulated from their storage state. Researchers from the lab of Professor Paula Hammond in the Department of Chemical Engineering at the Massachusetts Institute of Technology, describe a microneedle‐based platform that enables the simultaneous storage and delivery of dengue virus vaccine in a single formulation/system. Using well‐known excipients such as maltodextrin, trehalose, and poly(vinylpyrrolidone) alongside a layer‐by‐layer templating approach to coat the surface of microneedles, the authors show that suitable storage of dengue virus could be achieved for up to 3 weeks. Importantly, dengue virus was stored directly on the surface of the microneedle delivery system, which the authors used to successfully demonstrate protection against wild type dengue virus. Collectively, this work demonstrates the usefulness of a single system that can both enable long‐term storage of vaccines and simultaneously deliver vaccine payloads capable of protecting against viral challenges.

DOI: 10.1002/btm2.10127

